# A Review of Signaling Transduction Mechanisms in Osteoclastogenesis Regulation by Autophagy, Inflammation, and Immunity

**DOI:** 10.3390/ijms23179846

**Published:** 2022-08-30

**Authors:** Xishuai Tong, Gengsheng Yu, Xiaohui Fu, Ruilong Song, Jianhong Gu, Zongping Liu

**Affiliations:** 1 Institutes of Agricultural Science and Technology Development (Joint International Research Laboratory of Agriculture and Agri-Product Safety of the Ministry of Education of China)/College of Veterinary Medicine, Yangzhou 225009, China; 2 Jiangsu Co-Innovation Center for Prevention and Control of Important Animal Infectious Diseases and Zoonoses, Yangzhou 225009, China; 3 Jiangsu Key Laboratory of Zoonosis, Yangzhou 225009, China

**Keywords:** osteoclastogenesis, autophagy, inflammation, immunity

## Abstract

Osteoclastogenesis is an ongoing rigorous course that includes osteoclast precursors fusion and bone resorption executed by degradative enzymes. Osteoclastogenesis is controlled by endogenous signaling and/or regulators or affected by exogenous conditions and can also be controlled both internally and externally. More evidence indicates that autophagy, inflammation, and immunity are closely related to osteoclastogenesis and involve multiple intracellular organelles (e.g., lysosomes and autophagosomes) and certain inflammatory or immunological factors. Based on the literature on osteoclastogenesis induced by different regulatory aspects, emerging basic cross-studies have reported the emerging disquisitive orientation for osteoclast differentiation and function. In this review, we summarize the partial potential therapeutic targets for osteoclast differentiation and function, including the signaling pathways and various cellular processes.

## 1. Introduction

Bone renewal and remodeling are dynamic courses that include old bone removal by osteoclasts and new bone generation by osteoblasts [1]. Bone also provides mechanical assistance to the body, locomotion, and protects the internal organs, such as the brain, lung, kidney, and bone marrow [2]. Typically, bone maintains a normal metabolic equilibrium. However, excessive osteoclast activity or osteoblast depletion causes degenerative joint- and bone-related diseases, such as osteoporosis and osteolytic Paget disease [3,4]. In contrast, attenuating osteoclast activity and/or osteoclast dysfunction or reinforcement osteoblast remodeling causes abnormal bone density-related diseases, such as osteopetrosis and osteogenesis imperfecta [5,6].

**Figure 1:**
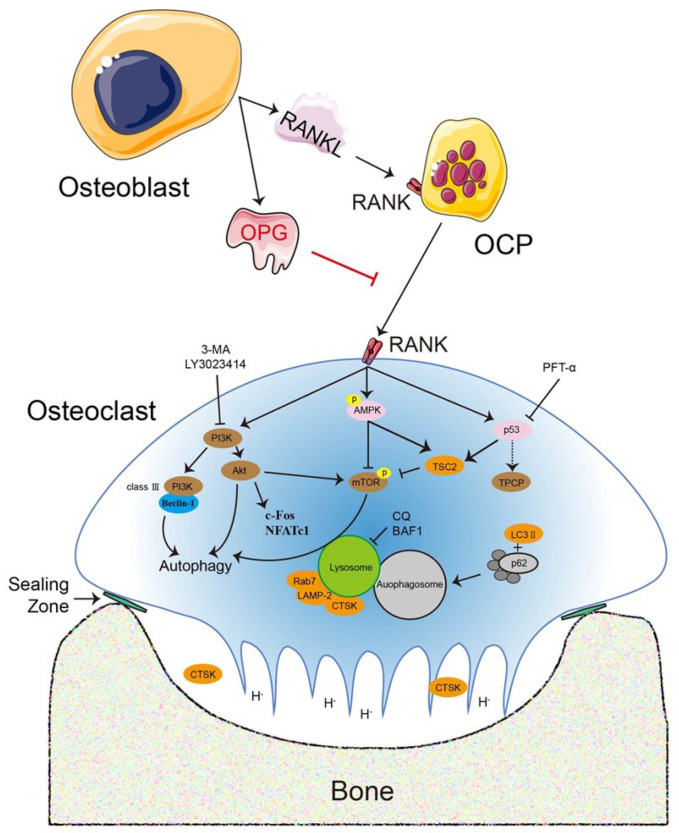
The RANKL–RANK–OPG system is a key regulatory axis that is indispensable during osteoclastogenesis and is regulated by numerous signaling pathways and dependent or independent routes. RANKL and OPG are secreted by osteoblasts or other cells and maintain the dynamic equilibrium for bone metabolism, which includes bone matrix degradation and regeneration mediated by osteoclasts and osteoblasts, respectively. The association with osteoclastogenesis is required for RANKL to bind to its receptor RANK, whereas OPG suppresses osteoclastogenesis. Various signaling pathways or regulators directly or indirectly regulate osteoclastogenesis, such as the PI3K–Akt and AMPK–mTOR signaling pathways, p53, and the transcription factors c-Fos and NFATc1. An exogenous secreted factor, OPG inhibits osteoclast differentiation and function directly. Accordingly, the role of various critical regulators in osteoclastogenesis has been examined with PI3K inhibitors [3-methyladenine (3-MA) and LY3023414], the p53 inhibitor pifithrin-α (PFT-α), and lysosomal inhibitors [chloroquine (CQ) and bafilomycin A1 (BAF1)].

Osteoclasts are multinucleated terminal giant cells regulated by two essential cytokines: macrophage colony-stimulating factor (M-CSF or CSF-1) and receptor activator of nuclear factor κB (NF-κB) ligand (RANKL). M-CSF is commonly secreted by bone marrow stromal cells (BMSCs) and osteoblasts and is critical in osteoclastogenesis biological function. M-CSF binds to its cognate receptor c-Fms (on monocyte/macrophage and osteoclast surfaces) and promotes osteoclastogenesis [2,7]. M-CSF promoted RANKL-induced osteoclast survival and motility, whereas incomplete blockade of c-Fms attenuated bone loss and did not prevent ongoing osteoclastogenesis. Nevertheless, M-CSF cannot be considered as irreplaceable and indispensable as RANKL. RANKL combines with receptor activator of NF-κB (RANK) on the surface of osteoclast precursors (OCPs) or mature osteoclasts and simultaneously activates downstream cascade signals to accelerate osteoclastogenesis [8,9]. Osteoprotegerin (OPG) is a secretory glycoprotein produced by osteoblasts or BMSCs. OPG competitively combines with RANKL to block RANKL binding to RANK, thereby inhibiting osteoclastogenesis [10,11,12]. The RANKL–RANK–OPG system is a well-known key regulatory axis and is indispensable during osteoclastogenesis [9,13,14] (Figure 1).

Although this regulatory mechanism was identified in previous osteoclastogenesis studies, understanding of the mechanism is limited. Generally, many aspects affect osteoclast differentiation and function, which involves the regulatory mechanism of OCP migration and fusion, osteoclast secretion, and the exogenous action of other cells. Therefore, this review highlights the role of osteoclastogenesis signaling transduction in the interaction with autophagy, inflammation, and immunity and proposes their relationship for future applications in bone cell differentiation and function.

## 2. Master Regulators of Osteoclastogenesis

Osteoclastogenesis requires a series of transcription factors and degrading enzymes including M-CSF, which is secreted by osteoblasts or BMSCs and is a survival and proliferative factor [15,16]. Similarly, RANKL indirectly and/or directly stimulates osteoclastogenesis by regulating the phosphatidylinositol 3-kinase (PI3K)–protein kinase B (Akt) signaling pathway, AMP-activated protein kinase (AMPK), and p53, lysosomal-associated regulators, the transcription factors nuclear factor of activated T cells c1 (NFATc1) and activator protein-1 (AP-1), and OPG [17].

PI3K is a lipid kinase that contains three subfamilies (class I, II, III). Class I PI3K is critical for cell survival, differentiation, proliferation, and metabolism [18]. A lack of class I PI3Kα (p85α) in mouse bone marrow monocytes/macrophages (BMMs) led to the reduction of activated Akt and phosphatidylinositol-3,4,5-trisphosphate (PIP3) levels, which is related to M-CSF binding to its tyrosine kinase receptor c-Fms [19,20]. In mice, class 1 PI3Kγ (p110γ) knockout resulted in augmented bone mass by decreasing osteoclast numbers and impairing osteoclast formation in vivo and in vitro, but did not affect bone formation [20].

The role of class II PI3K is unclear, especially in cell signaling transduction. Class III PI3K is mainly involved in regulating vesicle trafficking, including autophagy, phagocytosis, and endocytosis [21]. Class III PI3K combines with Beclin1 to form a complex that initiates autophagy, thereby affecting the regulation of downstream signaling [22,23]. These actions were suppressed by the PI3K inhibitor 3-methyladenine (3-MA), which caused the phosphorylation of mammalian target of rapamycin (mTOR) and p70S6K during osteoclastogenesis [8].

Akt has a positive regulatory function in RANKL-induced osteoclastogenesis by increasing NFATc1 expression but does not affect the expression of c-Fos and the inactive form of GSK-3β [24]. Akt contains three members: Akt1, Akt2, and Akt3. Akt1 and Akt2 are expressed in similar locations, such as bone, fat, liver, and muscle. Akt3 is limited to the testis and brain and was not expressed on BMMs and osteoclasts [25,26,27,28]. Mice with double-elimination of Akt1 and Akt2 exhibited loss of bone mass and development injury and even died shortly after birth [29]. The major Akt1 isoform is present in bone cells (osteoblasts, BMMs, osteoclasts), where its deficiency in mice caused decreased bone mass and formation by impairing osteoblast and osteoclast function [27]. Importantly, each isoform performs different functions that cannot be replaced and compensated by another [30,31]. Recent, a novel inhibitor, LY3023414, was examined in the PI3K–mTOR signaling pathway in a phase Ⅱ clinical study, where it suppressed Akt1 and Akt2 expression in osteoblastogenesis [25]. These data suggested that the PI3K–Akt signaling pathway is critical in bone cells, especially RANKL-induced osteoclastogenesis.

AMPK is a critical sensor in regulating cellular energy metabolism in eukaryotes. AMPK contains three subunits: α, β, and γ. AMPKα subunit phosphorylation at Thr172 is induced by decreased ATP/AMP and the activation of calcium/calmodulin-dependent protein kinase kinase β (CaMKKβ) and liver kinase (LKB1), which are the two upstream kinases [32,33,34]. An important monitor in response to the balance of cellular ATP level, AMPK is coupled with its functional homeostasis in regulating mitochondrial function, autophagy, and glucose metabolism [35]. In the presence of a physiological concentration of ATP (5 mmol/L) in mammalian cells, AMP led to the increased allosteric activation of both the γ1 and γ2 complexes by approximately 10-fold, whereas γ3 complex activation became almost negligible (1.4-fold higher) [36]. CaMKKβ and LKB1 are potential activators in bone cells. Phosphorylation of CaMKKβ promotes osteoclast differentiation and function. Otherwise, it has an inhibitory effect. In this section, we mainly focus on the relationship between AMPK and osteoclast differentiation and function.

AMPK plays a negative role in osteoclastogenesis by regulating autophagy [32,37,38,39]. The AMPKα isoform has a catalytic action in osteoclast differentiation and function via signaling cascades. The activation of AMPKα stimulated OPG secretion from osteoblasts and bone mineral density and indirectly attenuated the differentiation and function of osteoclasts induced from RAW264.7 cells to alleviate bone loss [40]. Continuous activation of the AMPKα2 isoform enhanced osteogenesis while attenuating M-CSF and RANKL secretion and the RANKL/OPG ratio to result in lower osteoclastogenesis [39]. In contrast, inactivation of AMPKα at Thr172 promoted osteoclast differentiation and function regulated by CaMKK and TGF-β-activated kinase 1 (TAK1) in vitro [41]. An in vivo study reported results consistent with those previous results, where osteoclasts caused decreased bone mass [42]. The AMPK regulatory β and γ subunits are critical in bone cells. Deletion of the AMPKβ isoform did not affect the osteoclast number but reduced trabecular bone mass and density [43,44]. In MC3T3 cells (osteoblastic cell line), activation of the AMPKβ isoform at Ser108 by prostaglandin E2 (PGE2) was an important mediator of the arachidonic acid derivative and promoted OPG synthesis [43]. The AMPKγ subunit consists of isoforms γ1, γ2, and γ3, which have different functions in osteoclastogenesis. Silencing of AMPKγ activity using knockdown or inhibitor (dorsomorphin) decreased its expression and osteoclast size but had no effect on osteoclast number [45,46].

More studies have focused on osteoblast mediation by p53, which indirectly controls osteoclastogenesis. p53 is a primary tumor suppressor that is also a negative regulator of bone remodeling with multiple regulatory mechanisms in osteoclastogenesis and osteoblast-dependent osteoclastogenesis [47]. p53 is related to osteoblast differentiation and has a dual role in regulating bone mass, where p53 deficiency in mice enhanced bone mass by increasing osteoblast differentiation while accelerating osteoclastogenesis that may have been induced by M-CSF secretion [47,48,49]. p53 is also regulated by the tumor suppressor ARF that controls ribosomal biogenesis and is involved in bone remodeling, including osteoblast function and osteoclast activity independently of p53 in vivo [50]. Interestingly, ARF inhibited cell proliferation in a p53-dependent or -independent manner, while its deletion in vivo enhanced osteoclastogenesis and bone resorption [51].

Previous data demonstrated that p53 accelerates osteoblastic differentiation driven by the transcription factors runt-related transcription factor 2 (RUNX2) and Osterix [47,52]. RUNX2 is a master regulator in BMSC-induced osteoblast differentiation induced [53]. RUNX2 was highly expressed in p53-deficient mouse embryonic fibroblasts, which had higher alkaline phosphatase (ALP) activity induced by bone morphogenetic protein 4 (BMP4) than normal fibroblasts [54]. Deletion of *Runx2* in mouse primary osteoblasts impaired osteoblastogenesis and the expression of major bone matrix proteins while inhibiting osteoclastogenesis due to the decreased RANKL secretion in osteoblasts [55]. RUNX2 promoted osteoclastogenesis induced by RANKL-stimulated RAW264.7 cells, whereas its mutation led to the loss of osteoclast differentiation and function [56]. Osterix is a vital transcription factor involved in early-phase osteoblastogenesis derived from osteoblast precursors [47,57]. The Osterix zinc finger region interacts with the p53 DNA-binding domain under Osterix and p53 overexpression. However, this interaction was downregulated by p53-mediated Osterix transcriptional activity during osteoblast differentiation [58].

As mentioned above, there are few reports on how p53 directly regulates osteoclastogenesis. p53 deficiency in BMSCs greatly promoted serum OPG production, resulting in increased bone density and trabecular (and not cortical) bone thickness. This was manifested as damaged osteoclast differentiation induced by p53-deficient monocytes, suggesting that p53 negatively regulates osteoclastogenesis [57]. OPG acts as a decoy receptor of RANKL to repress osteoclastogenesis [59]. Previously, we demonstrated that p53 has a positive role in OPG-inhibited osteoclastogenesis in vitro and the inhibition of p53 activity by pifithrin-α (PFT-α) reversed this process [48].

Bone tumor diseases caused by different undefined reasons have an unusual relationship with p53 and osteoclast differentiation. Growth factor independent 1 (*GFI1*) expression was upregulated in multiple myeloma cells, while silencing *GFI1* in p53 wild-type, p53 mutant, and p53-deficient multiple myeloma cells led to apoptosis. Conversely, *Gfi1* overexpression in mouse multiple myeloma cells enhanced bone destruction by increasing osteoclast number and size, thereby promoting tumor growth [60]. There was a reciprocal inhibitory effect between translationally controlled tumor protein (TCTP) and p53 [61,62]. TCTP expression was elevated in osteoclastogenesis, while silencing TCTP led to suppressed osteoclast differentiation induced by human bone marrow cells (hBMCs) [63].

### 2.1. Lysosome-Associated Regulators

Osteoclasts transport different physical substances through membrane-bound intermediaries to resorb the bone matrix. Membrane trafficking involves the transportation of solutes and proteins and is performed by other eukaryotic cell macromolecules (the endocytic, secretory, and transcytotic pathways) [64]. In this process, the secretory lysosome is a specialized lysosome-related organelle in osteoclasts that represents the primary activation and warehouse sites for acid hydrolases, such as cathepsin K (CTSK) [65]. Osteoclastic lysosome-associated proteins participate in the degradation of bone matrix from the ruffled border (RB) in osteoclastic resorptive lacunae, including lysosome-associated membrane protein 1/2 (LAMP1/2) and the small Ras-related GTPase Rab7. In this review, we focus on how osteoclastic functional factors regulate osteoclastogenesis.

A cathepsin family member, CTSK is a lysosomal predominant protease and the only subtype expressed in osteoclasts [66,67]. CTSK is indispensable for bone resorption, resides in the lysosomes and cytoplasmic vesicles of osteoclasts, and is released to the bone matrix surface regulated by an integrin α_v_β_3_-induced sealing zone with bone [67,68]. In mice, *Ctsk* knockout led to osteopetrosis and development of pycnodysostosis characteristics by decreasing osteoclastic resorptive activity and impairing CTSK secretion, which impeded bone organic matrix degradation and osteoclasts [69]. Deletion of the *Ctsk* gene in mouse hematopoietic cells or monocyte lineage cells reinforced bone volume, the bone formation rate, and osteoclast and osteoblast numbers [70]. In contrast, *Ctsk* overexpression recovered osteoclastic bone resorption in UTU17^+/+^ background mice, which also enhanced osteoblast formation by increasing ALP activity and soluble RANKL (sRANKL) secretion [71]. Trabecular bone volume was reduced in *Ctsk*-overexpressing transgenic mice, yet it accelerated osteoblast numbers, the bone turnover rate, and the amount of mineralizing surface [72].

CTSK is expressed abundantly in osteoclasts, replacing the other cathepsins to resorb the bone matrix [73,74]. CTSK is secreted from the lysosomes of mature osteoclasts and interacts with the E3 ubiquitin ligase Cb1 (Tyr737) adaptor protein and the PI3K p85 regulatory subunit [75]. A22, a specific potent inhibitor of CTSK, blocked osteoclast activation in vitro and enhanced the spine bone density in zebrafish [76]. In clinical tests, a series of pharmaceutical products were applied to inhibit CTSK activity, such as balicatib (AAE581), ONO-5534, and odanacatib, which were mostly efficacious for inhibiting osteoclast bone resorption and preventing bone loss. Nevertheless, these inhibitors were discontinued due to their adverse effects outside bone in clinical trials [66]. Angiogenesis-coupled osteogenesis was enhanced by treatment with the angiogenic factor platelet-derived growth factor (PDGF)-BB secreted from periosteal OCPs, especially for repairing fractures [77]. These studies suggested CTSK as a potential target for treating impaired osteoclastogenesis associated with bone-related diseases in vivo or in vitro.

LAMP is a crucial lysosomal membrane protein for phagosome fusion with lysosomes, has two isoforms (LAMP1 and LAMP2), and represents approximately 50% of the total amount of lysosomal membrane proteins [78]. LAMP2 is a key regulator of RANKL-induced osteoclastogenesis [78]. In osteoclasts, LAMP2 was enriched at the RB within actin rings while CTSK was transported into the resorptive lacuna with actin rings [79]. LAMP2 colocalized with Rab7 in mouse osteoclasts, but knockdown of Rab7 disrupted osteoclast polarization and the RB vesicles, which impaired bone resorption in vivo and in vitro [80,81]. Rab7 is a secreted lysosomal protein related to late endosomes/lysosomes and phagosomes that is localized to the osteoclast RB [64,82]. Rab7 has two GTP-dependent effectors [Rac1 and pleckstrin homology domain-containing protein family member 1 (PLEKHM1)], which are involved in the adaption of endosomal-lysosomal systems [83]. A Rab7-binding partner, Rac1 belongs to the small GTPase Rho family. Rab7 overexpression upregulated Rac1 activity, but silencing Rab7 inactivated Rac1 [84]. PLEKHM1 is present in osteoclast vesicles, but PLEKHM1 mutation or deletion was associated with the loss of function of osteoclast bone resorption caused by lysosomes and microtubules [79,83,85].

### 2.2. NFATc1 and c-Fos

NFATc1 is a master regulator responsible for osteoclastogenesis by regulating a series of downstream specific genes, such as that for CTSK, tartrate-resistant acid phosphatase (TRAP), c-Fos, and calcitonin receptor (CT) [86]. In the presence of RANKL, NFATc1-deficient embryonic cells failed to differentiate into osteoclasts, which suggested that NFATc1 is indispensable in osteoclastogenesis [87,88]. Conversely, NFATc1 overexpression reversed osteoclastogenesis in *Fos*-deficient mice [89]. In BMMs, NFATc1 overexpression induced osteoclastogenesis by enhancing the levels of the vacuolar ATPase V0 domain d2 isoform (Atp6v0d2) and dendritic cell (DC)-specific transmembrane protein (DC-STAMP) in the absence of RANKL and increased TRAP, CTSK, c-Src, and integrin α_v_β_3_ expression [90]. Cyclosporin A (which blocks *NFAT* transcription) inhibited osteoclastogenesis in the presence of M-CSF and RANKL by downregulating the expression of osteoclastic-specific factors (Atp6v0d2 and DC-STAMP) in vitro [90]. These data suggested that NFATc1 is critical in osteoclastogenesis.

A *Fos* gene family member, c-Fos is essential in osteoclastogenesis [91]. RANKL binds to its receptor RANK in osteoclast precursors or the osteoclast surface and recruits the tumor necrosis factor receptor-associated factors (TRAFs) to activate downstream signaling [89,92]. TRAF6 binds to RANK to mediate c-Src, Akt, and PI3K activation [93]. *Traf6*-deficient mice exhibited osteopetrosis caused by reduced osteoclast numbers [94]. Likewise, osteoclast formation in *Fos* (*c-Fos*)-deficient mice failed due to the reduced RANK expression in macrophages, which resulted in osteopetrosis [91]. Interestingly, *Fos* overexpression upregulated RANK expression but RANK overexpression failed to rescue RANKL-induced osteoclastogenesis in *Fos*-deficient mice [91]. In BMMs, c-Fos overexpression rescued RANKL-stimulated osteoclastogenesis by increasing NFATc1 expression [95].

MicroRNAs (miRNAs) represent post-transcriptional regulators of protein-encoding genes and are important during osteoclastogenesis [96]. RANKL stimulated c-Fos activation by upregulating miR-21 expression during osteoclastogenesis, which resulted in the downregulation of programmed cell death 4 (PDCD4) levels [97]. miR-145 mediated the cooperation between the c-Fos complex and Smad3 in osteoclastic-specific precursors, which reduced phosphorylated Smad2/3 complex formation and suppressed c-Fos and NFATc1 transcription [98]. miR-20a attenuated hypoxia-induced osteoclastogenesis by reducing autophagy-related 16-like 1 (ATG16L1) and microtubule-associated light chain protein 3II (LC3II) expression and the levels of osteoclastic-specific markers (NFATc1, TRAP, TRAF6) [99]. These studies demonstrated that c-Fos regulates transcription factor and signaling pathway activity in RANKL-induced osteoclastogenesis.

### 2.3. OPG

A soluble receptor for RANKL, OPG inhibits osteoclast differentiation and bone resorption by decreasing osteoclast numbers and RANK signaling in vivo and in vitro [100]. OPG is a transmembrane protein that promotes bone remodeling [11,101]. *Opg* transgenic mice exhibited severe but non-lethal osteopetrosis via decreased osteoclast number and bone resorption [12]. OPG-deficient mice exhibited alveolar bone loss via reduced sclerostin levels and accelerated bone resorption [102]. Conversely, the high bone density phenotype caused by *Opg* overexpression in transgenic mice resulted in suppressed osteoclastogenesis [12]. OPG inhibited osteoclast adherent structure differentiation and formation and correspondingly attenuated the expression of RANK, TRAP, integrin β_3_, matrix metalloproteinase-9 (MMP-9), and carbonic anhydrase I (CAII), which are involved in autophagy [103]. Previously, we demonstrated that OPG inhibited the expression of c-Fos (an AP-1 family member), CTSK, and NFATc1 in a dose-dependent manner in osteoclastogenesis [8]. These data indicated that OPG is a potential target for inhibiting osteoclastogenesis in various regulatory mechanisms (Figure 1).

## 3. Autophagy Regulates Osteoclastogenesis

Eukaryotic cells feature three types of autophagy: macroautophagy, microautophagy, and chaperone-mediated autophagy (CMA). Macroautophagy (usually termed autophagy) is a conservative survival pathway that can use abandoned intracellular materials for cellular energy production [104]. Recent studies demonstrated that autophagy is an intercellular conservative mechanism for removing misfolded proteins, damaged organelles, and macromolecules [105,106]. During autophagy in eukaryotic cells, a total of 32 ATGs were tightly related to LC3 and the adaptor molecule sequestome 1 (SQSTM1)/p62 [107]. Autophagy is a vital intracellular regulatory mechanism associated with osteoclastogenesis [108,109,110]. Clearly, osteoclast differentiation and osteoclast-mediated bone resorption are highly complex processes in bone remodeling and are involved in regulating the cell signaling pathways, transcription factors, and external environment. The role of AMPK on autophagy in osteoclastogenesis has been described previously [32]. Therefore, in this review, we discuss how partial regulators mediate osteoclastogenesis and are involved in autophagy.

Many studies demonstrated that autophagy regulation of osteoclastogenesis involves multiple cellular signaling pathways, such as that of PI3K–Akt–mTOR, AMPK–mTOR–ribosomal protein S6 kinase beta-1 (S6K1), and p53 [8,47,110,111,112]. In the initial stage of autophagy, autophagy activation and stimulation are caused by signaling factors at amino acid sites, such as the phosphorylation of class III PI3K and Unc-51-like autophagy-activating kinase 1/2 (ULK1/2) [113,114]. Activated PI3K causes Akt phosphorylation at different amino acid residue sites. For example, Akt Ser473 is involved in regulating the mTOR complex and Akt Thr308 is activated partially for full Akt phosphorylation [115]. PI3K is part of the PI3K complex that includes Beclin1, Vps34, UVRAG, and Ambra1 [22].

M-CSF binds to its receptor c-Fms to promote OCP survival and proliferation and phosphorylate four tyrosine residues (Y559, Y697, Y721, Y921), causing its autophosphorylation and transphosphorylation [116,117,118]. c-Fms phosphorylation at Y559 and Y721 interacted with *c-Src* to form a complex to activate the PI3K–Akt signaling pathway [119]. In primary osteoclasts, excessive dexamethasone induced bone loss caused by PI3K–Akt–mTOR signaling pathway-mediated elevation of autophagy [120]. We previously reported that AMPK–mTOR–S6K1 signaling pathway-mediated autophagy was involved in OPG-inhibited osteoclastogenesis in primary osteoclasts and osteoclast-like cells (OLCs) [8]. These data suggested that the PI3K–Akt signaling pathway has a vital effect on regulating autophagy during osteoclastogenesis.

mTOR contains two distinct complexes: mTORC1 and mTORC2. mTORC1 includes mTOR, regulatory-associated protein of mTOR (Raptor), mammalian lethal with SEC13 protein 8 (mLST8 or G protein beta subunit-like [GβL]), and the non-core components proline-rich Akt substrate of 40 kDa (PRAS40) and DEP domain-containing mTOR-interacting protein (DEPTOR). mTORC2 includes mTOR partner, rapamycin-insensitive companion of mTOR (Rictor), GβL, and mammalian stress-activated protein kinase (SAPK)-interacting protein (mSin1) [121,122]. mTORC1 is a switch for osteoclastogenesis proliferation-to-differentiation, which is a critical role that causes bone loss by increasing osteoclast activity at low-dose rapamycin treatment or inactivating mTORC1 during RANKL-induced osteoclastogenesis via the calcineurin–mTORC1–NFATc1 signaling pathway [123]. Likewise, rapamycin or mTOR small interfering RNA treatment inhibited osteoclast differentiation and bone resorption [16,124]. However, deleting mTOR or Raptor significantly inhibited osteoclastogenesis in BMMs derived from mTOR flox/– and Raptor flox/flox mice in vitro, and in vivo research yielded analogous results [125,126]. Conversely, late Raptor deletion, but not Rictor deletion, in osteoclast precursors enhanced osteoclastogenesis in mice [123,127].

mTOR activity is regulated negatively by upstream tuberous sclerosis complex 1 (TSC1). Deletion of the *Tsc1* gene in mouse bone marrow macrophages prevented osteoclast bone resorption and bone loss in vitro and in vivo [127]. TSC2 is associated with TSC1, forming the TSC complex, which negatively regulates mTOR mediated by the small G-protein Ras homologue enriched in brain (Rheb) [128,129]. Subsequently, the mTOR complex is activated by upstream Akt signaling, which causes p70S6K phosphorylation and the suppression of 4E-binding protein 1 (4E-BP1), leading to cell survival, proliferation, and metabolism [130,131]. p70S6K is an mTORC1 activity indicator that correspondingly decreased during osteoclastogenesis [132,133]. These studies demonstrated that mTOR-regulated autophagy is a major regulator in osteoclastogenesis.

Beclin1 is involved in initiating autophagy, which promoted the formation of autolysosomes from double-membrane autophagosomes fusing with lysosomes [134]. Beclin1 is indispensable for initiating autophagy in osteoclastogenesis [108], is pivotal in autophagy, and is enhanced in RANKL-stimulated osteoclastogenesis in vitro. 3-MA inhibition of autophagy suppressed osteoclast formation and resorption, accompanied by decreased Beclin1 and ATG5 expression [8,135]. Mice lacking Beclin1 exhibited increased cortical bone thickness caused by impeded osteoclast function and ATG-mediated autophagy in vivo [136]. Mice with Beclin1 knockout had increased cortical femoral bone mass but reduced trabecular bone quality and mass [108]. Conversely, lentiviral overexpression of Beclin1 stimulated autophagy, which increased osteoclastic bone resorption [137]. Beclin1 overexpression reversed osteoclastogenesis and autophagy deficiency in OCPs incubated with SP600125 (a JNK inhibitor), which resulted in suppressed autophagy and augmented apoptosis [138]. Beclin1 also plays a non-autophagic role and its expression was increased in a p38- and NF-κB-dependent manner during RANKL-induced osteoclastogenesis in vitro [139].

Osteoclastogenesis also requires the involvement of ATGs and autophagy-related proteins in bone resorption, then completes the degradation of old bone matrix with the specific zone. Beclin1, LC3, ATG5, ATG7, and ATG12 expression is upregulated in RANKL-induced osteoclastogenesis [140]. In autophagy, ATGs are essential for the degradation of intracellular useless cargos, and ATG7 aggregates the soluble forms of LC3I into LC3II, complementing normal cellular function [137]. The ATG5–ATG12 complex acts as a part of autophagosomes and prolongs phagocytic ring formation in the cytoplasm. Subsequently, the unnecessary molecules are transported to autolysosomes through ubiquitin p62 binding to LC3 [141]. The deletion of *Atg5* or *Atg7* suppressed osteoclast differentiation and impaired CTSK secretion respectively, leading to reduced osteoclast bone resorption [142,143]. Knockdown of p62 attenuated the formation of TRAP-positive osteoclasts and the F-actin ring, osteoclast-related gene expression, and LC3 accumulation [140]. p62 mutant mice had increased osteoclast numbers, which led to bone mass loss [144]. Based on these findings, ATGs have a more important regulatory role in osteoclastogenesis involving autophagy and the latter inversely regulates the former.

A negative regulator of bone metabolism, p53 can enhance osteoblast-dependent osteoclastogenesis and osteoclastic bone resorption by increasing M-CSF levels [47]. p53 negatively regulates osteoclastogenesis and is involved in the cell cycle and programed cell death, such as autophagy [48]. p53 activates autophagy, which is part of the p53 protective function, causing cell survival [145]. Activated p53 increased AMPKα phosphorylation by inhibiting mTOR-induced autophagy [146]. Autophagy has positive effects on tumor progression when p53 is activated, whereas autophagy is a part of tumor suppression when p53 function is lost [48,147]. Conversely, autophagy suppresses p53 and is important for tumor promotion. Previously, we demonstrated that p53 is critical during OPG-inhibited osteoclastogenesis by regulating TSC2-induced autophagy in vitro [48].

The lysosomal specific inhibitors chloroquine (CQ) and bafilomycin A1 (BAF1) reduce lysosome acidification by increasing the pH, which leads to mTOR activation and augments p62 levels to inhibit osteoclastogenesis while attenuating fusion and multinucleation [148,149]. mTORC1 activation requires a complex formed by the lysosomal membrane structure vacuolar-type H^+^-ATPase proton pump (V-ATPase). Correspondingly, BAF1 inhibited lysosomal activity in epiphyseal chondrocytes but potently caused mTORC1 signaling activation and p70S6K phosphorylation [150]. We demonstrated that CQ and BAF1 impaired osteoclast differentiation and function in the presence or absence of OPG [10]. In this process, inhibiting lysosomal function resulted in failure to bind to autophagosomes, but CQ increased autophagosome numbers and size, which promoted AMPK phosphorylation [10]. These data suggested that lysosomal regulators are critical in osteoclastogenesis by regulating autophagy.

## 4. Inflammation and Immunity Mediate Osteoclastogenesis

**Figure 2:**
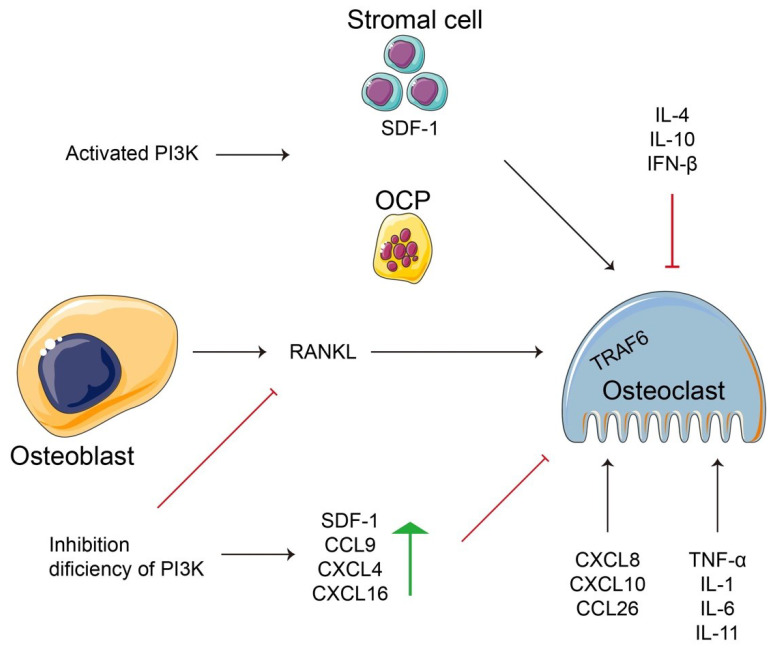
A schematic diagram of the relationship between inflammation and immunity for regulating osteoclastogenesis or osteoblast-dependent osteoclastogenesis. PI3K regulates the cell types (stromal cells, OCPs, osteoblasts, osteoclasts) to control cellular events, such as the release of anti- and/or pro-inflammation factors. Additionally, a few inflammatory factors not only implement their basic function according to their own definition, but are also involved in regulating various cells, such as osteoclastogenesis, or fused from OCPs.

Recently, there has been evidence regarding bone cell inflammation and immunity, which includes bone resorption and bone remodeling, especially the interaction between bone marrow immune cells and osteoclasts [151,152,153]. Osteoimmunity is an interdisciplinary concept originated by Arron and Choi on bone-related disease in 2000 [153]. Importantly, the RANKL–RANK–OPG system is a core element in osteoclastogenesis, providing a solid foundation in osteoimmunity [151]. Various immune regulatory cells (T and B lymphocytes, macrophages, DCs) were implicated in osteoclast-induced bone resorption [154]. Immune cells release proinflammatory cytokines, such as interleukin-1β (IL-1β), IL-6, tumor necrosis factor-α (TNF-α), and IL-11 and/or also secrete anti-inflammatory cytokines, such as IL-4, IL-10, and interferon-β (IFN-β), which are associated with the skeletal system. For example, IL-1 derived from the immune cells induced osteoclastogenesis [155] and TNF-α promoted osteoclastogenesis and bone resorption by activating autophagy in patients with rheumatoid arthritis in vivo and in vitro [156]. Osteoclastogenesis is related to the regulation of autophagy, inflammation, and immunity and is extremely complex (Figure 2).

The PI3K–Akt signaling pathway is a critical promotor of cellular proliferation, differentiation, and metabolism [157,158]. The pathway regulates osteoclastogenesis through various regulatory mechanisms as described in Section 2.1 and Section 2.2. Inhibition of the PI3K–Akt signaling pathway attenuated RANKL expression during osteoclastogenesis [159]. However, the activation of PI3K enhanced stromal cell-derived factor 1 (SDF-1 or CXC ligand 12 [CXCL12]) production and osteoclast precursor numbers in mouse bone marrow cells [160]. Osteoclast-mediated bone resorption is a vital process against excessive bone formation to maintain appropriate calcium levels in the blood [101]. Under pathological conditions, such as inflammatory conditions or estrogen deficiency, accelerated bone resorption results in osteolysis [161]. However, PI3Kγ-deficient mice had decreased osteoclast numbers following impaired osteoclast formation in vivo and in vitro, and increased levels of inflammatory chemokines (SDF-1, chemokine ligand 9 [CCL9], CXCL4, CXCL16) [20]. Chemokines are secreted dynamic molecules that are important regulators in osteoclastogenesis [162]. Recent studies have reported that CXCL8, CXCL10, and CCL20 contributed to osteoclastogenesis in response to changes in inflammatory molecules [163]. Moreover, CCL4 regulates osteoclast motility by promoting PI3K activity [162,164]. Therefore, the PI3K–Akt signaling pathway positively regulates immunity and inflammation in osteoclastogenesis.

The proinflammatory factors TNF-α, IL-1, and IL-6 have positive regulatory roles in response to autophagy in osteoclastogenesis [32,165,166]. In mice, TNF-α knockout inhibited osteoclast differentiation and function accompanied by suppressed autophagy [137]. In osteoclastogenesis, TNF-α induced transcriptional repressor B lymphocyte-induced maturation protein-1 (Blimp1) expression. Silencing Blimp1 in OCPs markedly attenuated TNF-α production in osteoclast differentiation [167]. Furthermore, TNF-α cooperated with IL-1 to activate functional osteoclast differentiation [168]. IL-1 induces osteoclast differentiation in the presence of RANKL through JNK activation, which is downstream of TRAF6 and has additive effects [169,170]. IL-1β promotes RANKL production and IL-6 synthesis from osteoblasts or osteocytes to further stimulate osteoclastogenesis [171,172]. Conversely, IL-4 stimulated OPG production by regulating the RANKL–RANK–OPG axis and inhibited RANKL-induced osteoclastogenesis in BMMs and RAW264.7 cells [173]. IL-4 plays a negative role in osteoclastogenesis and functions by inhibiting NF-κB signaling and NFATc1 expression [174,175]. In bone metastasis of colorectal cancer, IL-4 promoted the proliferation of early OCPs by binding its receptor IL-4α, while IL-4 deficiency impaired bone resorption [176]. In TNF-α-activated stromal cells, IL-4 suppressed TNF-α-mediated osteoclast formation by inhibiting RANKL expression and directly inhibited TNF-α-activated osteoclast precursors in vivo via a T cell-independent mechanism [177]. IL-4 also acted with IL-10 to negatively regulate osteoclastogenesis by reducing NFATc1 expression, and IL-10 deficiency promoted bone loss in mice [178]. Therefore, inflammation may be required for osteoclast formation and function and can be modulated by different factors, such as chemokines, cytokines, and hormones.

The immune cell and the bone cell are tightly linked with osteoclast differentiation and function. Among them, the interaction between immune cells and osteoclasts in the bone marrow or joint cavity is the basis of osteoimmunity [151]. For example, activated T lymphocytes promotes osteoclastogenesis via producing RANKL [179]. However, overexpression of RANKL in T lymphocytes of RANKL-deficient mice can restore osteoclastsogenesis [151,180]. In addition to RANKL, the type 17 helper T (Th17) cell-derived IL-17 has long been recognized as a crucial pre-inflammatory cytokine that facilitate osteoclastogenesis [155,181,182]. IL-17 is a complex of inflammatory cytokines indirectly involved in osteoblast-induced osteoclastogenesis, and binding of the IL-17 receptor complex augmented bone mass through conditional deletion of IL-17a receptor (IL-17ra) in osteoclast precursors [183]. Similarly, low IL-17 concentrations increased RANKL-induced osteoclastogenesis via Beclin1–TAK1-binding protein 3 (TAB3)–extracellular signal-regulated kinase (ERK) pathway-mediated autophagy, but high IL-17 levels had the opposite effect [184]. In addition, monocytes or tissue-specific macrophages (macrophages resident in tissues) are a major bone cells, which is origin of osteoclasts in inflammatory and immune environment [151]. Macrophages represent a type of myeloid monocyte with phagocytic properties, and they are mainly involved in the inflammatory response and immune response to keep dynamic balance [185]. Osteoclastogenesis fused with osteoclast precursors, which are derived from monocyte-macrophages [186]. Several identified genes are directly involved in macrophages membrane fusion, including CD9, CD81, CD44, and CD47 [187,188,189]. CD9 and CD81 negatively regulate macrophages fusion into osteoclast differentiation, while CD44 promotes the osteoclastogenesis by the of NF-kB/NFATc1 signaling pathway, and CD47 also stimulates the fusion of osteoclast precursors [189,190]. In summary, immune cells and bone cells cooperate to execute the effect of osteo-related immunity, which is shared by a common microenvironment and regulatory molecules.

## 5. Conclusions and Perspectives

Osteoclastogenesis is an extremely complicated regulatory process that includes fusion between OCPs and cytokine-induced differentiation and is controlled by other regulatory factors. These actions involve two essential cytokines (M-CSF and RANKL) to control osteoclastic bone resorption. More evidence has demonstrated that osteoclastogenesis originated from the diversification of cell species, such as monocytes/macrophages and DCs. In vitro studies have the advantage of being able to focus on the regulatory mechanisms of osteoclast formation and function, but they are also subject to more restrictions. First, multiple regulators and cascade signaling pathways control osteoclastogenesis, revealing the intricacy of differentiation and function in vivo. Second, osteoclasts are responsible for bone resorption and are involved in different intracellular metabolisms, including autophagy, apoptosis, inflammation, and immunity. Finally, a certain regulator of osteoclastogenesis may participate in ≥1 regulatory modes or courses to perform functions other than its own. This may make it easier to control osteoclastogenesis equilibrium by either internal regulation or external stimulation.

Inflammation and immunity are intricately linked to bone-related diseases and interact and influence each other. The deepening and richness of bone mechanism research demonstrates not only the involvement of blood immunity mediated by inflammatory cytokines, but also presents the considerable challenge of the different bone cell regulatory contents. For example, recent studies concentrated on the positive role of inflammatory cytokines on osteocytes, including osteoblasts, osteoclasts, OCPs, and DCs, but might have neglected the effects of negative regulators. Nevertheless, we have only described the role of a few cytokines in osteoclastogenesis stimulated by different cells, such as OLPs or other cells with which they interact.

Lastly, endogenous hormones exert a critical effect on osteoclastogenesis by controlling inflammation and immunity and other factors, such as PGE2, parathyroid hormone, progesterone, estrogen, and vitamin D. These regulators feature large and convoluted mechanisms of action in the animal body, including bone. In particular, the bone marrow contains abundant cells and interacts with the blood, which acts on the tissues and organs and is susceptible to environmental or drug influences.

## Data Availability

The data that support the findings of this study are available from the corresponding author upon reasonable request.
